# On the Limitations of Using Ribosomal Genes as References for the Study of Codon Usage: A Rebuttal

**DOI:** 10.1371/journal.pone.0049060

**Published:** 2012-12-20

**Authors:** Ruth Hershberg, Dmitri A. Petrov

**Affiliations:** 1 Rachel & Menachem Mendelovitch Evolutionary Processes of Mutation & Natural Selection Research Laboratory, Department of Genetics, Faculty of Medicine, Technion-Israel Institute of Technology, Haifa, Israel; 2 Department of Biology, Stanford University, Stanford, California, United States of America; American University in Cairo, Egypt

## Abstract

In a recent paper published in PLOS ONE, Wang *et al.* challenge our finding that the identity of optimal codons in different genomes follows a set of clear rules. Here we provide a rebuttal of their paper and demonstrate that the results of our original PLOS Genetics paper stand. This provides us with an opportunity to bring up an aspect of how codon usage has been studied that should be of general interest. The Wang *et al.* study, as well as many other studies, used ribosomal genes as a reference set for the study of patterns of codon usage. We discuss here the assumptions that are made in order to justify using ribosomal genes to study codon bias, suggest that this practice can at times be problematic, and discuss its limitations.

## Introduction

A complex combination of natural selection, mutation, and random forces such as genetic drift and draft shapes the nucleotide sequences of genomes. It is often difficult to distinguish when selection affects certain genome features, and when the effects of selection on a given feature are demonstrated it is even harder to elucidate the causal mechanisms that lead to this selection. Nowhere is this more the case than when it comes to the subject of codon usage (reviewed in [1], [2]).

It has been known for a long time now that certain synonymous codons are consistently used at higher than expected proportions within the protein-coding genes of any given organism. This phenomenon has been termed codon bias (reviewed in [1], [2]). The nucleotide content of the genome at large is explained by background substitution patterns that are comprised of a combination of the effects of mutation (which we and others have recently demonstrated to be universally AT-biased [3], [4]), and of selection, or selection like processes such as biased gene conversion. Codon bias cannot be explained solely by these background substitution biases. Following decades of study and debate, it is currently well accepted that a selective pressure (separate from any selection involved in determining the background substitution biases of a genome) affects codon usage in all bacteria, in insects, and even in mammals (reviewed in [1], [2]).

Why does selection affect codon usage? This question is far from fully answered. The two most often cited, non-mutually-exclusive reasons for selection on codon usage are that by using certain codons it is possible to increase on the one hand the efficiency of translation and on the other hand its accuracy. Strong evidence exists that selection to increase translation accuracy affects codon usage from bacteria to mammals (for example see [5]–[8]). Evidence for selection to increase translation efficiency at this point is not as strong [1], [2]. Both of these types of selection are expected to affect more strongly genes that are highly expressed. Selection for translation efficiency should affect highly expressed genes more strongly, because if such genes are not translated efficiently it can lead to increased ribosomal sequestering. This in turn would hinder the translation of all of the genes within the genome. Selection for translation accuracy is expected to affect highly expressed genes more strongly, because if such genes are mistranslated a larger proportion of the translated proteome will have errors that can affect protein function and folding. This can result in the loss of function of a larger proportion of the proteome, and, perhaps even more severely, lead to misfolded protein aggregation, which can cause cell death. While selection to increase translation accuracy and efficiency are the most well-studied, it is very likely that selection acts on codon usage for additional, less well characterized reasons [1], [2], [9]. For example, codon usage can be affected by the need of transcripts to have particular mRNA structures, by the presence of regulatory binding sites within transcripts, and in the case of many organisms by the need of transcripts to be properly spliced [10], [11]. While it is unclear whether these factors will affect more strongly highly expressed genes, it does seem likely that groups of genes that carry a particular function and/or belong to a particular gene family will be more similarly affected by these factors than genes at large.

Those codons within each genome that are favored by a global selective force, such as the need for optimal translation, have been named “favored” or “optimal” codons. We recently identified the favored codons in 675 bacteria, 52 archea, and 10 fungi [12]. Through this analysis we found that across all studied organisms the identity of favored codons tracks the GC content of the genomes. In organisms in which background substitution biases drive nucleotide contents to be GC-rich, favored codons will also be GC-rich. The opposite will be true for organisms in which background substitution biases are towards AT. Once the effect of the genomic GC content on selectively favored codon choice was taken into account, additional, universal, amino acid specific rules governing the identity of favored codons became apparent [12].

The method we used to identify favored codons was to ask which of the codons encoding a particular amino acid increase in frequency as genes become more biased in the choice of codons overall. Following Wang *et al.* [13], we will from now on refer to this as the *correlation method*. This methodology offers the advantage of making as few assumptions possible as to nature of selection on codon usage, and as to the identity of the genes that are under such selection. All that is assumed when using the correlation method is that genes that are more biased relative to the local background substitution patterns are under stronger selection to increase codon bias, than genes that are less biased. In addition, in order to be able to identify favored codons using the correlation method, the identity of those codons that are favored by selection to optimize translation, or by any other global selective pressure acting on codon usage needs to be consistent across genes. Otherwise, the correlation method will likely fail to identify any codons as favored.

An alternative approach is to rely on ribosomal genes as a reference set for the identification of favored codons. Following Wang *et al*. [13], we will from now on refer to this approach as the *comparison method*. Under this approach, favored codons are identified by comparing the codon usage between ribosomal genes and the rest of the genome. Several assumptions need to be made in order to use this method. One assumption is that ribosomal genes will be highly expressed, and therefore will be under strong selection to use favored codons, across all organisms. This seems to be quite reasonable. We have previously demonstrated that in 658 of 675 bacterial genomes tested, ribosomal genes are statistically over-represented among the 100 most codon-biased genes within the genome [12]. The second key assumption is that differences in codon usage between ribosomal and non-ribosomal genes that are due to differences in selection acting on translation accuracy and/or efficiency (or due to any other kind of genome wide selective pressure, that affects highly expressed genes more strongly) are significantly more pronounced than differences due to other selective constraints *specific to* ribosomal genes. As we discuss in this paper, although this might often be the case there are cases where this assumption is clearly violated and thus should not be made as a rule.

This article is written, first and foremost, as a rebuttal of an article that has recently been published by Wang *et al.* in PLOS ONE [13]. In this article Wang *et al.* use the comparison method to identify favored codons, and challenge our previous findings about the general rules that govern favored codon choice. Here we demonstrate that some of the results they obtained using the comparison method are clearly false, and use this as an opportunity to discuss the possible limitations of the extremely common practice of using ribosomal genes as a reference in studying codon usage. In addition we provide a point-by-point rebuttal of the claims made in the Wang *et al.* paper, about our results, and therefore demonstrate that our original results stand (Box S1).

## Results and Discussion

### Using Ribosomal genes as a reference for identifying favored codons can lead to clearly false conclusions

**Figure 1:**
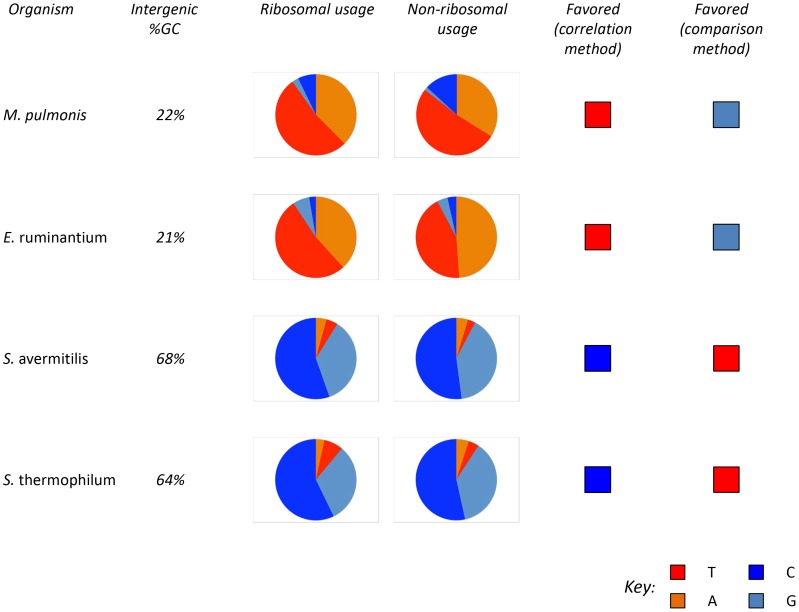
Favored codons identified using the comparison method are often incorrect. We present examples of the frequency distributions of all Alanine codons in ribosomal and non-ribosomal genes, in four randomly selected bacterial genomes in which there is a disagreement regarding the codon identified as favored between our study (which used the correlation method) [12] and the study of Wang *et al.* (which used the comparison method) [13]. Alanine is a four-fold degenerate codon family, meaning the first two bases of the codon always remain the same. The data presented in this figure therefore focuses on the 3^rd^ codon position, which in the case of Alanine can be either A, or T, or G, or C. The results demonstrate that codons identified using the comparison method (as identified by Wang *et al.*), are always so rare, and that their enrichment in ribosomal genes is so un-substantial that it does not appear likely that they are in fact favored.

The main finding of our original study was that the identity of favored codons follows the background substitution biases of a given genome [12]. This means that for bacteria in which background substitution biases are towards GC (reflected by GC-rich intergenic regions), favored codons will tend to be GC-rich, while for bacteria in which background substitution biases are towards AT, favored codons will tend to be AT-rich. Wang *et al*. claimed that our results are incorrect, because when using the comparison method they sometimes identified AT-rich codons as favored in GC-rich genomes and vice versa. The example they highlighted was that of Alanine, a four-fold degenerate codon family. The way they performed the test was as following: For each synonymous codon family the frequency of each possible codon was calculated in the ribosomal genes and in all other genes and then the optimal codon was identified as the one showing the most significant increase in frequency from the non-ribosomal to ribosomal genes [13]. In Figure 1, we present examples of the frequency distributions of all Alanine codons in ribosomal and non-ribosomal genes, in four randomly selected bacterial genomes in which there is a disagreement regarding the codon identified as favored between our study and the study of Wang *et al*. It is immediately apparent that the codons identified by Wang *et al*. are in fact very unlikely to be favored. For example in the GC-rich bacterium *Streptomyces avermitilis* Wang *et al.* identify the codon GCT as favored, because it encodes 4.7% of the Alanines present in ribosomal genes and only 3% of those present in non-ribosomal genes. Such enrichment, while apparently statistically significant, is clearly not indicative of the GCT codon being favored by selection. After all the GCT codon is remarkably infrequently used across all genes within the genome, including ribosomal genes that are supposedly highly expressed. It is far more likely that such a small difference will either be stochastic, or be due to some more specific, weak constraint acting on ribosomal genes, but not on non-ribosomal genes as a whole. Such results are not limited to the four randomly selected bacteria presented in Figure 1. Wang *et al.* most often identify GCT as the Alanine favored codon. This is the case both in GC-rich and GC-poor genomes. Yet, when one examines GC-rich genomes (>60% GC content in intergenic regions), GCT will only encode on average 8.7% of Alanines in ribosomal genes, and 4.5% of Alanines in all other genes in the genome. It is also interesting to note that in all the cases examined, the codons we identified as favored also increased in frequency between ribosomal and non-ribosomal genes (Figure 1).

The Alanine example was highlighted in the Wang *et al.* paper as demonstrating that the comparison and correlation methods sometimes identify different favored codons. Wang *et al*. claimed this as evidence that codons identified by the correlation method were sometimes incorrect, and that our finding that favored codon choice follows genomic GC content was therefore also incorrect. Above, we provide evidence that in those cases in which different Alanine codons were identified as favored by the two methods, the codons identified by Wang *et al.* are actually extremely unlikely to be favored. More interesting to the general reader, these results provide an example of why it may problematic to use ribosomal genes (or any other group of genes) as a reference for the identification of favored codons.

The Wang *et al.* paper made a number of additional claims that attempted to discredit our results. Our full rebuttal of all their claims is given in Box S1.

### Patterns of codon usage of non-ribosomal genes are measurably affected by selection

We have provided above and in box S1 a rebuttal of the Wang *et al.* paper. We would now like to take this opportunity to discuss the much more widespread practice of using ribosomal genes as a reference in the study of codon bias, and raise some possible concerns with this wide-spread practice.

In the Wang *et al.* paper favored codons were identified by comparing codon usage between 37 ribosomal genes, and 3 elongation factors, that were implicitly assumed to be the only highly expressed genes within any given genome, and the reminder of genes within the genome, which were assumed to all be lowly expressed [13]. (A direct quote from the paper reads: “Following the previous studies, a total number of 40 genes, including 3 elongation factor genes and 37 ribosomal protein genes, are regarded as high expression genes in a genome and all the remaining genes can be reasonable thought as low expression ones”). This leads to an assumption that any difference in codon usage between ribosomal and non-ribosomal genes will be due to selection acting to increase translation accuracy and/or efficiency of ribosomal genes, but not of other genes. Any other possible reason for differences in codon usage between ribosomal genes and non-ribosomal genes is therefore neglected. A somewhat softened version of this assumption is that the number of genes within each genome that are expressed at high enough levels to be under selection for codon usage is small. Thus, it is assumed that the combined effects of selection on all genes within a genome, excluding the ribosomal genes, is weak to the point that it does not affect patterns of codon usage to any measurable extent, and that the signal of selection on codon usage will be observed in the ribosomal genes but will not be observed when considering all the other genes together. (for example a quote from [14] reads: “*S* was estimated from the codon frequencies in a set of 40 genes expressed at very high levels compared with those in the genome as a whole, with the latter taken as an indication of the frequencies generated by mutation biases in the absence of selection.”). This version of the assumption has been used in order to estimate the strength with which selection on codon usage acts in different genomes, by comparing levels of codon bias between ribosomal genes and all other genes within a genome [14], [15].

If indeed ribosomal genes are the only genes highly expressed enough to be under selection for codon usage, or if they constitute the majority of such genes, to the point that selection on codon usage does not affect the remainder of genes in a measurable way, we would expect the distribution of levels of codon bias across genomes to reflect this. In other words, we would expect to find that very few genes within a genome are as biased in their codon usage as ribosomal genes, while the vast majority of the genes within a genome are not biased beyond what is expected from background substitution patterns. We examine this by carrying out two types of analyses. First, we ask how many genes within each genome are at least as biased as the average ribosomal gene (Materials and Methods). We find that this varies greatly between genomes, from 1.1% of genes in *Leuconostoc citreum* KM20, to 75.2% of genes in *Thermofilum pendens* Hrk5. The average percentage of genes that are more biased than the average ribosomal gene across all genomes is 17.3% and the median is 14.6%. This demonstrates that in many genomes a substantial number of genes are as or more biased than the average ribosomal gene. Thus the assumption that ribosomal genes are the only genes under selection for codon usage, or that very few other genes are under such selection seems unfounded. Interestingly, a recent study by Supek *et al*. [16] in which a supervised machine learning approach was used to detect the effects of translational selection on codon usage, demonstrated similar results. Supek *et al*. used their approach to estimate the proportion of genes within each bacterial genome that are similarly biased to ribosomal genes. They report that, in the 461 prokaryotic genomes they examined, such genes constitute between 5% and 33% of all the genes, with an average of 13.2% [16].

**Figure 2:**
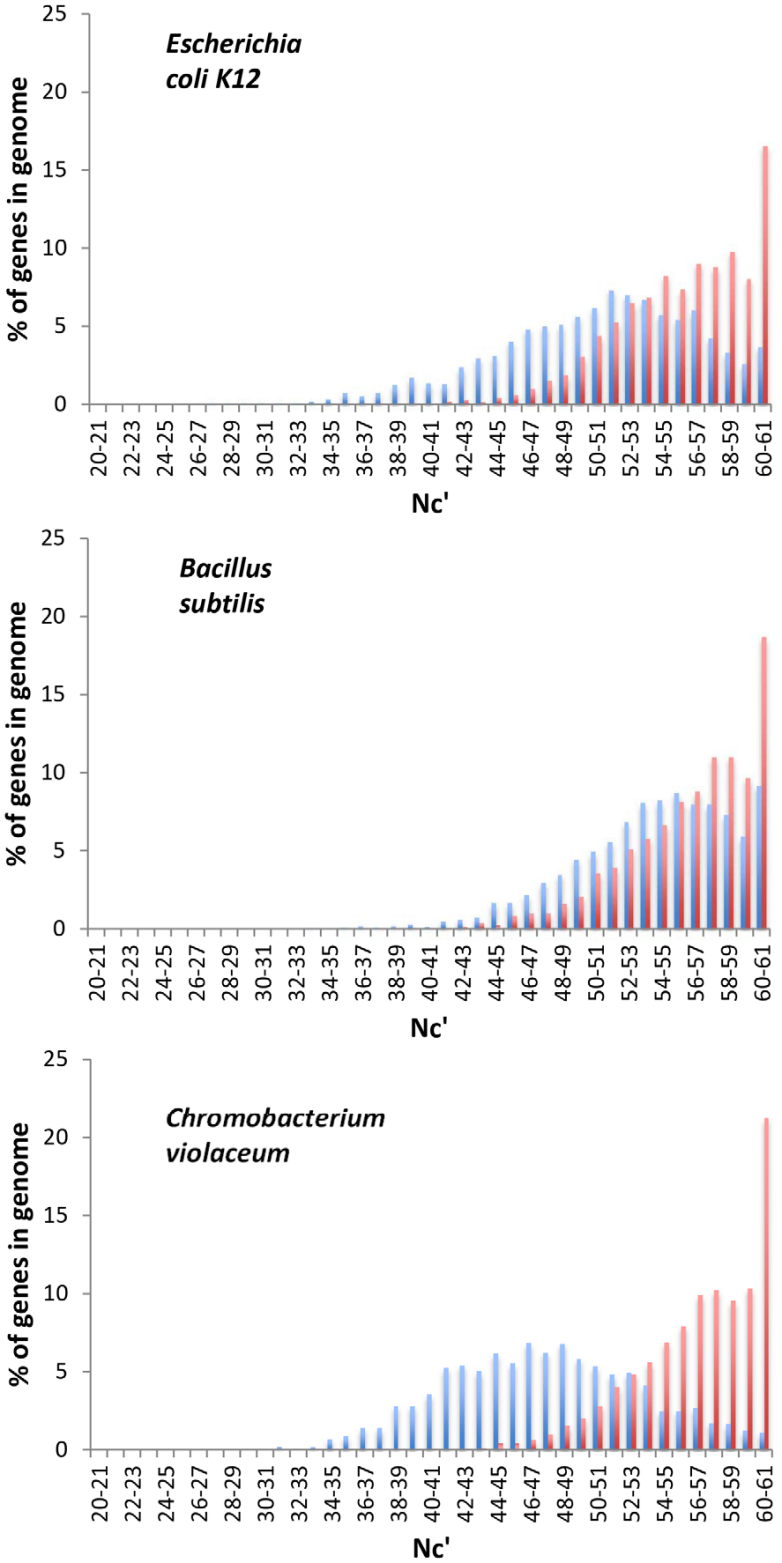
The number of non-ribosomal genes affected by selection on codon usage varies greatly between genomes. For three representative genomes the distributions of overall levels of codon bias of non-ribosomal coding segments (CS, Blue), and Intergenic Control Coding Segments (ICCS, Red) are presented. Codon bias is measured using Nc′, a measure of codon bias that ranges between 20, for extremely biased genes that use only one codon per amino acid, to 61, for genes that use all synonymous codons equally [19]. Levels of codon bias in ICCS should result from background substitution biases alone, while levels of bias within CS are the result of both background substitution biases and selection on codon usage, if such selection affects a given CS.

The second type of analysis we carried out is to ask whether the distribution of levels of codon bias suggests that only a small number of genes are under selection. For each genome the following analysis was performed: We extracted the first 100 fourfold and twofold degenerate codons from each protein coding gene (excluding ribosomal genes). We then replaced the third codon positions of these coding segments (CS) with 100 randomly selected nucleotides from the intergenic sequences adjacent to each CS, while maintaining the identities of the encoded amino acids. This resulted in a set of intergenic control coding segments (ICCS, see Materials and Methods). Levels of codon bias within ICCS should reflect the levels of codon bias expected to result from background substitution biases, in the absence of additional selection on codon usage. We can compare the distributions of levels of codon bias of CS and ICCS across a genome (Figure 2). The results presented in Figure 2 for three different bacterial genomes demonstrate that the number of non-ribosomal genes that have higher levels of codon bias than expected in the absence of selection varies greatly between genomes. Based on these results it appears that the number of genes in which selection affects codon usage may be higher in *Escherichia coli* compared to *Bacillus subtilis*, and may be higher still in *Chromobacterium violaceum*, where a majority of genes appear affected by selection on codon usage.

Differences in the proportion of genes that are under selection for codon usage between genomes may be the result of either differences in the numbers of highly expressed genes in each genome, and/or of differences in the level of expression needed within a given genome for a gene to be under selection to use favored codons. The former may stem from differences in the frequency of genes with different functional categories between genomes, as different functional categories may tend to be differentially expressed [16], [17]. The later can stem from differences in the long-term effective population sizes (N_e_) between genomes. In bacteria with larger N_e_ selection will affect synonymous mutations that have smaller fitness effects compared to genomes with smaller N_e_. In other words selection on codon usage (and on any other variation that carries a fitness effect) will be stronger when N_e_ is higher. Interestingly, a method that attempts to estimate the strength with which selection on codon usage affects a given genome by assuming that ribosomal genes are highly affected by such selection, while all other genes as a whole are unaffected, may deduce stronger levels of selection when fewer genes are affected by selection. This is of course not what is meant to be achieved by such an analysis.

### Comparing codon usage between ribosomal genes and other genes may result in the identification of traits that are specific to ribosomal genes, and not in the identification of genome-wide effects

The assumption made by Wang *et al.* was that ribosomal genes are the only highly expressed genes in the genome, and are thus the only ones that will be enriched for favored codons [13]. This is, as made clear above, a false assumption. While Wang *et al.* implicitly make this assumption (see quoted text above), such a strong assumption does not necessarily need to be made in order to use ribosomal genes as a reference for identifying favored codons. In order to use ribosomal genes to identify favored codons, it is however necessary to assume that differences in codon usage between ribosomal and non-ribosomal genes due to genome-wide selection are more pronounced than differences due to other ribosomal gene-specific selective constraints. Whether or not this assumption holds will depend partly on the extent to which ribosomal and non-ribosomal genes are differently affected by selection on codon usage in a given genome. We have demonstrated above that the proportion of non-ribosomal genes affected by selection on codon usage varies greatly between genomes. As discussed above the ability to use ribosomal genes as a reference for identifying favored codons will also depend on the extent to which ribosomal genes have specific traits affecting their composition. In some cases it may be possible that ribosomal specific traits will be more pronounced than differences in more global selective constraints that affect ribosomal genes as well as large portions of non-ribosomal genes.

**Figure 3:**
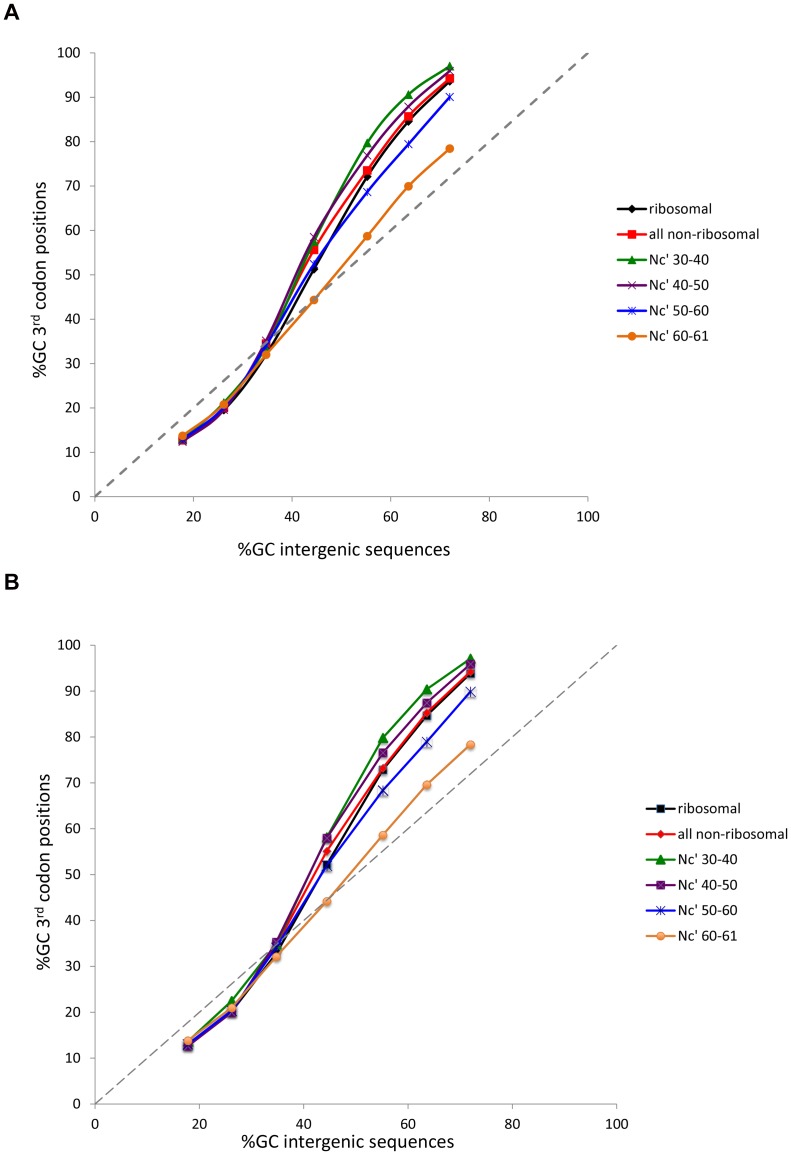
The “going with the flow” trend. In GC-rich genomes 3^rd^ codon positions are even more GC-rich than intergenic regions, and in AT-rich genomes the opposite trend can be observed. This “going with the flow” trend reflects the tendency of genomes in which background substitution biases are towards GC to use GC-rich favored codons, and the opposite tendency of genomes in which background substitution biases are towards AT to use AT-rich favored codons. The trend becomes stronger as levels of codon bias of genes increase (with reduced Nc′). Even though ribosomal genes on average have Nc′ values of 46+/−4, their trend is somewhat weaker than that of other genes with similar levels of codon bias. The trend for ribosomal genes is also slightly weaker than that of all other genes as a whole. To create these trend lines, bacterial genomes were binned in increments of 10% by their intergenic GC contents. Each point on the X-axis reflects the average intergenic GC content within the given bin. (A) GC content is calculated for the 3^rd^ codon positions of all codons. (B) GC content is calculated only for the 3^rd^ codon positions of codons encoding Alanine.

As stated above, we found that favored codons tend to be GC-rich in genomes where background substitution patterns drive nucleotide contents towards GC, and AT-rich in genomes where background substitution patterns drive nucleotide content towards AT [12]. As a result of this, in GC-rich genomes 3^rd^ codon positions are even more GC rich than intergenic regions, and in AT-rich genomes the opposite trend can be observed (Figure 3A). As expected, this trend becomes stronger with an increase in the overall level of codon bias of a gene (Figure 3A). In other words, genes that are more biased will use more favored codons. In GC-rich genomes such codons will be GC-rich, and so the 3^rd^ codon positions of such genes will be more GC-rich. The opposite will be true for AT-rich genomes. We have named this trend “going with the flow”, because selection on codon usage follows the flow of background substitution biases when it comes to “choosing” the identity of favored codons. Interestingly, this trend can be observed even for genes with the lowest levels of codon bias (Figure 3A), indicating that even among such genes there is some selection to use favored codons. This again points to the fact that it is unreasonable to assume that patterns of codon usage in non-ribosomal genes are not observably affected by selection. The going with the flow trend also further supports our finding that favored codons tend to be AT-rich in AT-rich genomes and GC-rich in GC-rich genomes.

The “going with the flow” trend seems to be slightly less strong (although still very strong) for ribosomal genes compared to other genes with similar levels of codon bias (Figure 3A). This finding is particularly striking in GC-rich genomes, where it is strongly statistically significant (P<<0.0001, n = 224, using a paired Mann-Whitney test). Of 224 genomes examined with intergenic GC contents of over 50%, 94% have lower GC content in the third codon positions of ribosomal genes, compared to other genes with similar levels of codon bias. To a lesser, yet still strongly statistically significant extent, the “going with the flow” trend also seems to be less strong in ribosomal genes when compared to all non-ribosomal genes (*P* = 0.0006). Thus, it is possible that when one compares the codon usage of ribosomal genes to that of all other genes, one will observe differences resulting from this slight difference in the trend, while ignoring the far more pronounced “going with the flow” trend in its entirety. This can lead to false conclusions. For example, in a recent study Hildebrand e*t al.* used 3^rd^ codon positions to estimate mutational biases [4]. As a control in their study, they wanted to demonstrate that GC-richness is not favored in 3^rd^ codon positions. To this end they compared the GC-richness of 3^rd^ codon positions in ribosomal genes to those of all other genes within the genome, and found (as expected from Figure 3A) that ribosomal genes tend to have lower GC content than non-ribosomal genes, even in GC-rich genomes. From this they concluded that selection actually favors AT in 3^rd^ codon positions rather than GC [4]. However, this completely ignores the fact that in both ribosomal genes and across the genome, 3^rd^ codon positions are extremely enriched for GC compared to intergenic sites (Figure 3A). Thus, it appears quite clear that, contrary to their conclusion, selection does prefer GC in the 3^rd^ codon positions of GC-rich bacteria.

To make certain the “going with the flow” trend presented in Figure 3A, and the slight difference in codon usage observed between ribosomal genes and non-ribosomal genes with similar levels of codon bias is not somehow due to differences in amino acid usage, we repeated the same analysis using only codons encoding a specific amino acid (presented for Alanine in Figure 3B), or only fourfold degenerate codons (Figure S1). In both cases results remained consistent with what we report above: (1) The “going with the flow” trend remains apparent, and increases in magnitude with overall level of codon bias of genes (Figure 3B, and Figure S1), and (2) For GC-rich genomes with intergenic GC contents higher than 50%, 3^rd^ codon positions of ribosomal genes are significantly less GC rich than 3^rd^ codon positions of other genes with similar levels of codon bias (*P*<<0.0001).

Our findings demonstrate that there are (at least in GC-rich bacteria) slight, yet highly statistically significant differences between the codon usage of ribosomal genes and other genes with similar levels of codon bias. Furthermore they demonstrate that these differences cannot be due to amino-acid usage differences since the significance is maintained when one focuses on only Alanine codons, and/or on only fourfold degenerate codons. We argue that the slight difference observed between ribosomal and non-ribosomal genes when it comes to the extent of the “going with the flow” trend stems from specific constraints applied to ribosomal genes due to the fact that they are a co-regulated, co-expressed, interacting and, often, homologous group of genes. When one contrasts patterns between different groups of genes one will sometimes identify what distinguishes these groups. However, when one looks across all genes within the genome, one will identify patterns that affect the entire genome. Selection to increase the efficiency and accuracy of translation is not expected to affect only ribosomal genes, and so when comparing ribosomal genes to all other genes we may also identify signals that are not associated with this global selection. What we will identify, at those instances, may be the specific traits of ribosomal genes compared to all other genes. That ribosomal genes share such specific traits that distinguish them from all other genes (including other highly expressed genes) is supported by the results we present in Figure 3, and from the fact that, at least in some genomes SVMs can be used to identify ribosomal genes, based on amino-acid and codon usage alone [18]. That differences between ribosomal and non-ribosomal genes are not due only to differences in amino acid usage is supported by the results we present in Figure 3B and in Figure S1. Namely, our finding that the “going with the flow” trend seems to be slightly less strong for ribosomal genes compared to other genes with similar levels of codon bias holds when amino-acid usage is controlled for (Figure 3B, and Figure S1).

### Concluding remarks

This paper has two objectives. First, and perhaps less interestingly to the general reader, we demonstrate that the results of the Wang *et al.* paper [13] that were presented as contradicting our previous findings [12] were in fact incorrect (see Box S1). Second, and of more general interest we raise the concern that the common practice of using ribosomal genes as a reference set for the study of codon usage, holds severe limitations and can often lead to erroneous results. We suggest that when one seeks to study patterns of codon usage by using ribosomal genes, one should first examine the assumptions made in order to justify this usage. If the question asked only requires one to assume that ribosomal genes are among those genes within a genome that are highly expressed and therefore expected to be under selection to increase translation accuracy and/or efficiency, such an assumption appears to be justifiable. However, if analyses require an assumption that ribosomal genes are the only genes within a genome exposed to selection to increase codon bias, that they constitute the majority of such genes, that across genomes they constitute a constant fraction of such genes, that all differences in codon usage between ribosomal and non-ribosomal genes will stem from differences in global selection to optimize translation, or that selection at the level of codon bias at non-ribosomal genes is negligible, then such assumptions are not always well-founded and can greatly affect the obtained results. In general, the point needs to be made that by contrasting patterns of codon usage between defined groups of genes (e.g. between ribosomal and non-ribosomal genes), one will zoom in on what distinguishes those different groups of genes. It is possible that at times what distinguishes between the gene groups will provide a stronger signal than the signal provided by differences in the intensity of selection at the level of translation optimization. In such cases using ribosomal genes as a reference for the study of codon usage may prove problematic. At the same time, if one examines patters across genes within the genomes it is possible to observe genome-wide patterns such as those expected to result from selection to increase translation accuracy and/or efficiency.

## Materials and Methods

### Calculating the overall codon bias of genes

To calculate the overall codon bias levels of a given sequence, we used the Nc′ measure of codon bias which was developed by Novembre [19]. This method is similar to the effective number of codons measure (Nc) [20], except that it corrects for the nucleotide content of genes (using the nucleotide content of each gene separately). Both Nc, and Nc′ measure the overall codon bias of a gene across all codon families. The measures do not make any assumptions regarding the identity of the favored codons. Values of Nc and Nc′ range between 20, for extremely biased genes that use only one codon per amino acid, to 61, for genes that use all synonymous codons equally.

### Creating sets of intergenic control coding sequences

To create the intergenic control coding sequences (ICCS) we used the following strategy for each studied genome. I) We extracted the first 100 four-fold degenerate and two-fold degenerate codons of each protein coding gene. We removed from consideration genes that had less than 100 two-fold and four-fold degenerate codons. II) For each protein coding gene we extracted its two adjacent intergenic sequences. We concatenated both adjacent intergenic sequences (the 5′ and the 3′ intergenic sequences) and selected a 100 base pair segment of this sequence at random. We shuffled these sequences randomly. We removed intergenic regions shorter than 50 bases and if for a gene there was not at least 100 bases of adjacent intergenic region, we removed that gene from consideration. We shuffled the order of the nucleotides of these intergenic segments randomly III) We created ICCS using the real coding sequences as a backbone and replacing the third codon positions, based on the shuffled adjacent intergenic sequences, while maintaining the encoded protein sequence. For example if in the real protein at the tenth position we have a Valine encoded by the four-fold degenerate codon GUG and the shuffled segment of the adjacent intergenic sequence has a T in the tenth position, our ICCS will have a GUT in the tenth codon position. In the case of a two-fold degenerate codon such as the Lysine codons AA(A/G), we selected AAG if the corresponding intergenic position contained either a G or a C and AAA if the corresponding intergenic position contains an A or a T.

At the end of this process we obtained for each genome two sets of coding segments of a consistent length; the “real” coding sequences (CS) and the ICCS. Both of these encode exactly the same proteins. The third codon positions of the ICCS reflect the composition of the real gene's adjacent intergenic regions.

## Supporting Information

Figure S1 **The “going with the flow” trend is maintained when GC content is calculated only for 3^rd^ codon positions of four-fold degenerate codons.** To create these trend lines, bacterial genomes were binned in increments of 10% by their intergenic GC contents. Each point on the X-axis reflects the average intergenic GC content within the given bin.
(TIF)

Box S1 **Detailed rebuttal of Wang** ***et al.*** **PLOS ONE paper: “Optimal codon identities in bacteria: implications from the conflicting results of two different methods.”**
(DOCX)
