# Supplementary figures and images for: On the Limitations of Using Ribosomal Genes as References for the Study of Codon Usage: A Rebuttal
Source: PLoS One. 2012 Dec 20;7(12):e49060. doi: 10.1371/journal.pone.0049060 (PMC3527481; doi:10.1371/journal.pone.0049060)

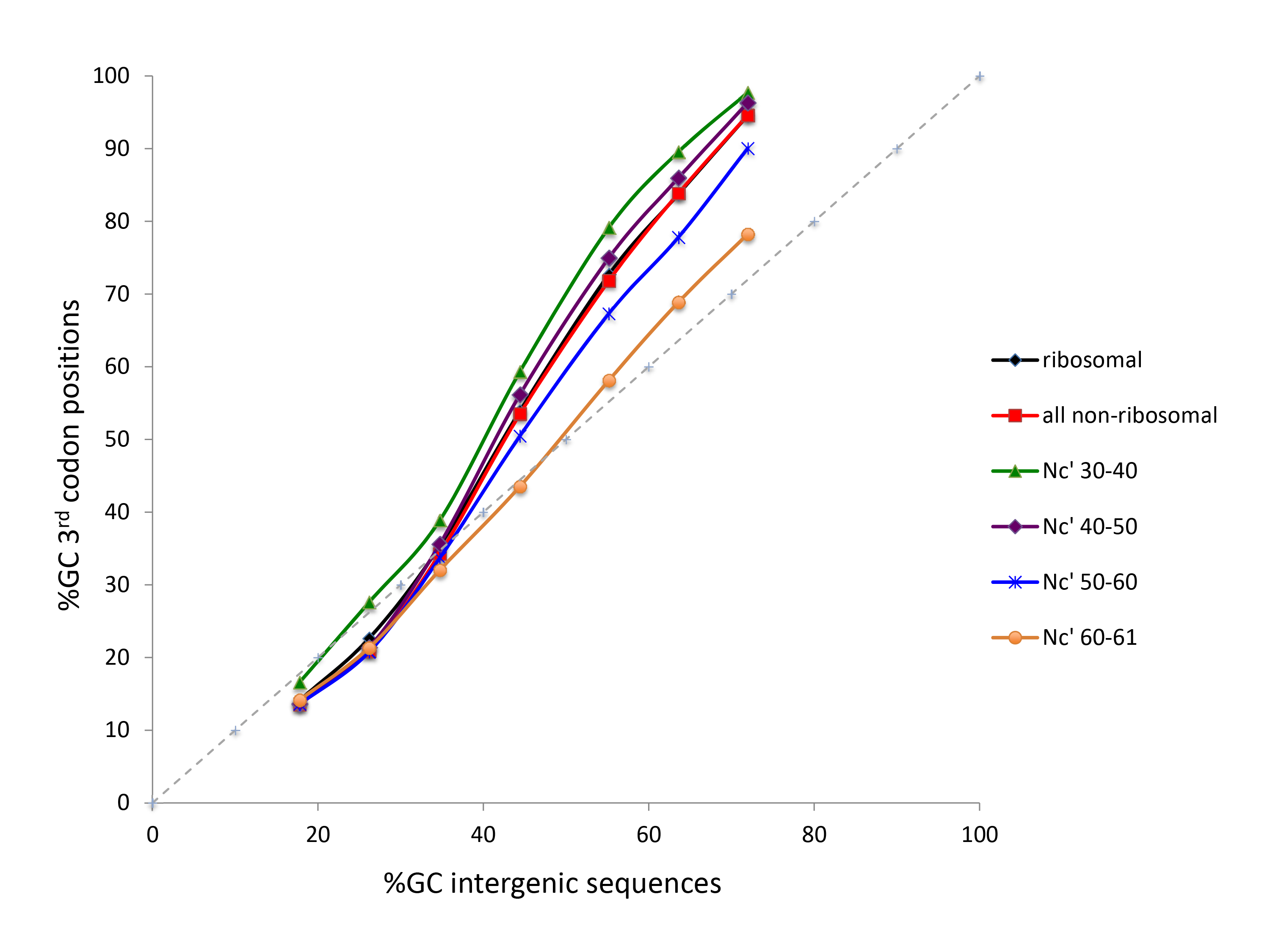

Supplement: Figure S1 — The “going with the flow” trend is maintained when GC content is calculated only for 3rd codon positions of four-fold degenerate codons. To create these trend lines, bacterial genomes were binned in increments of 10% by their intergenic GC contents. Each point on the X-axis reflects the average intergenic GC content within the given bin. (TIF) [file pone.0049060.s001.tif]
