# Supplementary material for: On the Limitations of Using Ribosomal Genes as References for the Study of Codon Usage: A Rebuttal
Source: PLoS One. 2012 Dec 20;7(12):e49060. doi: 10.1371/journal.pone.0049060 (PMC3527481; doi:10.1371/journal.pone.0049060)
Supplement: Box S1 — Detailed rebuttal of Wang et al. PLOS ONE paper: “Optimal codon identities in bacteria: implications from the conflicting results of two different methods.” (DOCX) [file pone.0049060.s002.docx]

**Box S1. Detailed rebuttal of Wang *et al.* PLoS One paper.**

Here, we go through the main points made by the Wang *et al.* paper in attempting to discredit our finding that the identity of favored codons follows a genomes background substitution patterns, and detail our objections to each point in the Wang *et al.* paper [1].

**Point 1:** **Codons identified using the comparison methods, used by Wang *et al.* sometimes differ from those identified using the correlation method we used.** Wang *et al.* suggest that the comparison method, by which favored codons are identified as those codons enriched in ribosomal genes compared to all other genes, is the more correct method to use, and that therefore any disagreement with the correlation method means that the favored codons we identified are incorrect [1]. This is the main claim of their paper. Above we demonstrate that results obtained using the comparison method as used by Wang *et al.* are in fact often clearly wrong. For example (see also Figure 1) when it comes to Alanine (the codon family used in the Wang *et al.* paper as the main example), Wang *et al.* most often identify GCT as the favored codon. This is the case both in GC-rich and GC-poor genomes. Yet, when one examines GC-rich genomes (> 60% GC content in intergenic regions) GCT will only encode on average 8.7% of Alanines in ribosomal genes, and 4.5% of Alanines in all other genes in the genome. It is highly unlikely that the codon that is favored by selection, because its use increases translation accuracy and efficiency will be so extremely rarely used. In the text above we also explain why the assumptions made by the comparison method are unfounded, and discuss how such small differences in the usage of rare codons between ribosomal genes and other genes are more likely to either be stochastic, or be the result of specific weak constraints on ribosomal genes, rather than the result of global, genome-wide selective forces such as selection to increase translation accuracy and efficiency.

**Point 2: When genomes with low tRNA copy numbers are removed from the analysis, some codon families (5 out of 18) no longer use AT-rich favored codons in AT rich genomes.** Wang *et al.* partition genomes into those with low tRNA copy numbers, and those with high tRNA copy numbers. They claim that genomes with low tRNA copy numbers will be under weak selection for codon usage, while genomes with high tRNA copy numbers will be under strong selection for codon usage [1]. We are not quite certain that the assumptions behind such a partition are sound, as it has recently been demonstrated that patterns of codon usage are measurably affected by selection, across virtually all bacteria [2,3]. Such a partition of genomes does however affect the number of AT-rich genomes that will be considered. More AT-rich genomes fall into the low tRNA copy number category, and as a result the high tRNA copy number category has only 23 AT rich genomes (GC content lower than 40%). Wang et al. applied the correlation method we used, separately on these two groups of genes, and claimed that when they look only at genomes with high tRNA copy numbers the trend we report by which AT-rich genomes have AT-rich favored codons, no longer holds. They base this on five codon families (Asp, Asn, His, Phe, and Tyr), in which G or C more often comes out as the favored codon, in the 23 AT-ich high tRNA copy number genomes, they analyzed. The tendency of these particular codon families to sometimes use G or C ending favored codons in AT-rich genomes has in fact been shown in our study as well (See Figure 6, and Supplementary Table 1 of our original paper). It is important to note, however, two facts: First, the five codon families highlighted are codon families for which in the majority of AT-rich genomes no favored codon can be identified (See Supplementary Table 1, and Figure 6 of our original paper). It is therefore hard to make conclusions based on these particular codon families. Second, and more importantly, even when considering only high tRNA copy number genomes, the majority of codon families (13 out of 18) do appear to use AT-rich favored codons in the AT-rich genomes, and GC-rich favored codons in the GC-rich genomes. This is much higher than expected by chance, and thus, the trend we observe, by which favored codons in AT-rich genomes tend to be AT-rich, remains unchanged.

**Point 3:** **Wang *et al.* find fault in the way we calculate Nc’** (a measure of the overall codon bias of a gene, that accounts for the nucleotide content of the gene in determining how biased it is beyond what would be expected by its nucleotide content alone) [4]. To support this, they make, to us, a puzzling and unclear set of claims and seem to imply that mutational biases are different within 3^rd^ codon positions compared to intergenic sequences. To make their point they show a figure that in fact presents the “going with the flow” trend we present here in Figure 3, and in Supplementary Figure 1. According to this trend 3^rd^ codon positions in GC-rich genomes will tend to be even more GC-rich than intergenic regions from the same genomes, while the opposite will be true for AT-rich genomes. The “going with the flow trend” is observed even when amino acid usage is controlled for (Figure 3B, Supplementary Figure 1). This trend is extremely unlikely to be the result of differences in mutational biases between genes and intergenic regions. In fact, we have demonstrated that mutation is AT-biased across bacterial genomes, and that this is true for both intergenic and genic regions [5]. What this trend in fact signifies is exactly the point we were making in our original paper [2]. Within genomes in which background substitution bias sequences towards GC, favored codons will tend to be GC-rich. As a result 3^rd^ codon positions will tend to be even more GC-rich than intergenic regions. The opposite will the true for AT rich genomes. It seems to us impossible to explain this trend without invoking selection on 3^rd^ codon positions to be GC-rich in bacteria with GC-biased background substitution patterns and AT-rich in bacteria with AT-biased background substitution patterns. Thus, when attempting to disprove our findings Wang *et al.* in fact provide evidence that our results are indeed correct.

**Point 4:**  **Wang *et al.* claim that the codons they identify using the comparison method better fit genomic tRNA compositions and are therefore more likely to be favored.** This analysis seems to assume that we know exactly how selection on codon usage in general, and how selection to increase the accuracy and efficiency of translation, in particular works, that we know all tRNA modification rules in all genomes, that we know how well expressed different tRNAs are in different genomes, and that we understand within each given genome which tRNAs and which codons best interact following modifications (after all, tRNA modifications have been shown to influence codon-anticodon preferences (e.g.[6-8])). In fact however, our knowledge of these factors remains far from complete, as becomes evident when reading the fascinating and diverse literature on this subject (for example see [3,9,10]). Thus, the analysis that Wang *et al*. claim shows that the codons they identify using the comparison method better fit tRNA composition is far from convincing.

**References**

1. Wang B, Shao ZQ, Xu Y, Liu J, Liu Y, et al. (2011) Optimal codon identities in bacteria: implications from the conflicting results of two different methods. PLoS One 6: e22714.

2. Hershberg R, Petrov DA (2009) General rules for optimal codon choice. PLoS Genet 5: e1000556.

3. Supek F, Skunca N, Repar J, Vlahovicek K, Smuc T (2010) Translational selection is ubiquitous in prokaryotes. PLoS Genet 6: e1001004.

4. Novembre JA (2002) Accounting for background nucleotide composition when measuring codon usage bias. Mol Biol Evol 19: 1390-1394.

5. Hershberg R, Petrov DA (2010) Evidence that mutation is universally biased towards AT in bacteria. PLoS Genet.

6. Meier F, Suter B, Grosjean H, Keith G, Kubli E (1985) Queuosine modification of the wobble base in tRNAHis influences 'in vivo' decoding properties. EMBO J 4: 823-827.

7. Grosjean H, de Crecy-Lagard V, Marck C (2010) Deciphering synonymous codons in the three domains of life: co-evolution with specific tRNA modification enzymes. FEBS Lett 584: 252-264.

8. Kruger MK, Pedersen S, Hagervall TG, Sorensen MA (1998) The modification of the wobble base of tRNAGlu modulates the translation rate of glutamic acid codons in vivo. J Mol Biol 284: 621-631.

9. Rocha EP (2004) Codon usage bias from tRNA's point of view: redundancy, specialization, and efficient decoding for translation optimization. Genome Res 14: 2279-2286.

10. Saks ME, Conery JS (2007) Anticodon-dependent conservation of bacterial tRNA gene sequences. RNA 13: 651-660.
